# A Novel Scheme for an Energy Efficient Internet of Things Based on Wireless Sensor Networks

**DOI:** 10.3390/s151128603

**Published:** 2015-11-12

**Authors:** Shalli Rani, Rajneesh Talwar, Jyoteesh Malhotra, Syed Hassan Ahmed, Mahasweta Sarkar, Houbing Song

**Affiliations:** 1 Research Scholar, Computer Applications, IKG Punjab Technical University, Kapurthala 144601, India; 2 ECE, Jhanjeri, Chandigarh 140307, India; E-Mail: rtphdguidance@gmail.com; 3 ECE, GNDU Regional Campus, Jalandhar 144001, India; E-Mail: jyoteesh@gmail.com; 4 School of Computer Science & Engineering, Kyungpook National University, Daegu 702-701, Korea; E-Mail: hassan@knu.ac.kr; 5 Electrical and Computer Engineering Department, San Diego State University, San Diego, CA 98182, USA; E-Mail: msarkar2@mail.sdsu.edu; 6 Department of Electrical and Computer Engineering, West Virginia University, Montgomery, WV 25136, USA; E-Mail: Houbing.Song@mail.wvu.edu

**Keywords:** Internet of Things, wireless sensor networks, clustering, energy consumption, transmission time

## Abstract

One of the emerging networking standards that gap between the physical world and the cyber one is the Internet of Things. In the Internet of Things, smart objects communicate with each other, data are gathered and certain requests of users are satisfied by different queried data. The development of energy efficient schemes for the IoT is a challenging issue as the IoT becomes more complex due to its large scale the current techniques of wireless sensor networks cannot be applied directly to the IoT. To achieve the green networked IoT, this paper addresses energy efficiency issues by proposing a novel deployment scheme. This scheme, introduces: (1) a hierarchical network design; (2) a model for the energy efficient IoT; (3) a minimum energy consumption transmission algorithm to implement the optimal model. The simulation results show that the new scheme is more energy efficient and flexible than traditional WSN schemes and consequently it can be implemented for efficient communication in the IoT.

## 1. Introduction

The Internet of Things (IoT) has been visualized as the communication and integration of smart objects (things). The dominance of IoT leads to a novel context of upcoming services and applications. Various objects such as mobile phones, sensors, Radio Frequency Identification (RFID) tags, *etc.* are components of the IoT, which are linked to the Internet via wireless and wired networks. To satisfy the various requirements of users, the smart objects can sense, gather and transmit data. Communication can take place between: (i) the objects themselves and (ii) objects and people. To gain insight into the various issues related to the real world physical processes, the IoT has been realized as a vital solution. The technological developments in the field of IoT have presented many implementation challenges. Sensed data can be sent in queried form or in a continuous way. This requires energy efficient communication among the sensor nodes. More objects are deployed in the IoT, that is why large amounts of power are consumed in the whole process, so green networking plays a crucial role in the IoT to reduce power consumption and operational costs, lessen pollution and emissions and make the most of surveillance and environmental conservation [1,2,3,4,5].

The realization of cost reductions to achieve green networking is the research objective of this paper. Many energy efficient schemes for WSN have been proposed in the recent past such as hierarchy [6,7,8,9], *ad-hoc* [10,11,12,13] and exact [14,15,16,17] ones, but these studies have not examined the arrangement of the objects in consideration of an energy efficient IoT. In this paper, we have investigated the cost effective arrangement of the objects to ensure an energy efficient IoT and put forward an innovative deployment scheme. Firstly a hierarchical framework model [1] is given for the deployment of the IoT. This introduces the scalability feature in the IoT and makes it more extensible. After that an optimization model is presented on the basis of the proposed framework and this model is energy efficient, which smoothens the progress on the way to a green IoT. Finally a minimum energy consumption chain-based cluster coordinator algorithm (ME-CBCCP) is executed, which uses cluster-based topology and a novel transmission algorithm for the optimization of energy parameters. We prove that this scheme is more lithe and efficient compared to traditional approaches for WSNs and it can be easily implemented in an energy efficient IoT. Our contributions in this paper can be summarized as follows:

(1) A hierarchical structure for placement of network components, that is objects/things in the IoT, is presented here. This structure has the scalability feature of the IoT to extend it up to any level. Direct communications between the relay nodes and sensor nodes at the cluster level, migrate the network load from local nodes to local relay nodes to provide energy efficient communication. Inter-cluster communication via cluster coordinators shifts the load from cluster heads (in a lower cluster) to the cluster coordinators (in upper clusters) thus enhancing the network lifetime.
(2) An optimization problem is considered for the proposed network structure in terms of load balance and energy consumption for implementation of an efficient and scalable IoT. Thus, we propose, ME-CBCCP under the influence of clustering topology to resolve the optimization dilemma. This strategy facilitates the implementation of an energy efficient (green) IoT.
(3) With extensive simulations on randomly deployed sensor nodes, the proposed scheme is validated in comparison to the traditional WSN schemes and found to be more favored for various applications of IoT.

Our technique to implement this scheme as follows: the area is divided into various known cells assumed as the clusters. All the clusters are the same in size. Communication within the clusters is possible through the relay nodes. Communication with the upper cluster is possible via CCOs in the upper layers. Chains are formed among the various clusters which start from the CH and go through the cluster coordinators situated in the upper clusters, so communication in the field can be categorized as: (1) between local nodes and CH; (2) communication between the CHs and CCOs; (3) between CCOs of the lower and upper clusters.

The rest of the paper is organized as follows: an overview of related work is given in Section 2 followed by a description of the framework model in Section 3. ME-CBCCP is presented in Section 4 and the results of different simulations are discussed in Section 5.

## 2. Related Work

A lot of research has already been reported for efficient communication in WSNs for the deployment of a green IoT [17,18,19,20,21], but little work is found concerning energy efficient communication for a scalable IoT. Routing protocols can be categorized into three types [22]: (i) energy efficiency-based; (ii) reliability and network operation-based and (iii) network operation-based.

A comparative study of clustering-based routing protocols [23,24] revealed that these protocols are an optimized solution for IoT applications. Many hierarchal protocols were proposed in past such as HEED [25], PEGASIS [26], CODA [27], HCR [28], SEP [29], EECHA [30], EECS [31], DWEHC [32], EEUC [33], PANEL [34], EB-PEGASIS [35], CCS [36], BCDCP [37], LEA2C [38], T-DEEC [39], EESAA [40], MODLEACH [41], Cross layer protocol [42], *etc.* which try to optimize the energy efficiency by the use of optimal cluster head selection, forming chains of nodes, by balancing the load on the clusters, *etc*. In these protocols, nodes have different functionalities or different roles so they are classified as normal nodes and cluster heads. The main objective of these protocols is to enhance the network lifetime. These routing algorithms are not suitable for IoT applications as they require extra time to form clusters and are not scalable and they introduce more complexity.

The most widely used network architecture for routing protocols is the tree-based one. In this type of routing all nodes transmit data to one node that is a base station (BS). Many existing solutions like E-CHtree [43] and multi-hop LQI [44] construct trees to route the data in a many-to-one pattern, but these solutions are not applicable for IoT applications like environment monitoring and coal mine goof applications. Other patterns required to be considered like many-to-many and one-to-many communication.

The discipline of meta-heuristic Evolutionary Algorithms (EAs) has also been utilized by several researchers to tackle cluster-based routing problems in WSN [45,46,47,48], but at the cost of a stability period and delay which cannot be avoided in IoT applications.

UCEB-CMF [49] has proposed as an unequal clustering and multipath algorithm with multi-hop communication can be the best solution for coal mine goof applications, but this protocol is not suitable for environmental IoT applications as the redundancy of data will be increased, and processing of the same data by many paths puts an unnecessary burden on the nodes.

To aggregate the data from different locations, sink mobility in a controlled manner is proposed by Koç *et al.* [50]. It is the best approach in terms of energy efficiency, but controlling the movement of the sink in the large scale network becomes difficult. A hybrid node scheduling based on efficient chain routing is proposed in [51]. Its routing strategy can be used for both event and time driven applications. It does not take into account continuous monitoring applications. The sink is considered at the center, which is considered the best case to gather the data, but some applications like border security surveillance cannot place the sink in the center, so this protocol requires analyzing the worst case, *i.e.*, when the sink is far away from the nodes. Multi-criteria objective function performs a decentralization of the clustering protocol [52,53] different from LEACH. CHs are elected in every round, but the election of the CHs again and again also consumes node energy. Shortest path selection by forming the minimum spanning tree [54] has proved an improvement over genetic-based algorithms, but this protocol does not pay attention to the fault tolerance, which is essential for the reliability of the protocol. Self-organized tree-based routing [55] performs better than HEED in terms of energy efficiency, but root nodes which communicate with all the nodes may be far away from some nodes which can affect the communication energy of the nodes. The energy balanced algorithm was proposed for water environment systems [56]. How the nodes will communicate in case of failure of some nodes is not taken into account in this protocol. The objective of all the above mentioned protocols is the development of cluster-based approaches to conserve energy, but they do not consider the essential feature, scalability for the IoT [57].

Flat routing protocols represent some solutions for IoT applications. REL [58], LABILE [59], AODV [60] and EEURP [61] proposed the new schemes for the IoT, but they do not consider the load balance, energy efficiency and scalability QoS metrics.

A survey of these protocols prompted us to develop a new framework for the IoT with a routing algorithm which could enhance not only the network lifetime, but also reduce the delay. Network lifetime can be enhanced by minimizing the communication distance of the nodes and by load balancing on the CHs. The connectivity of the network is also important to deliver the data in a timely way, but it is assumed in these protocols that the connectivity of the nodes can’t be maintained because of failure of the nodes and this may render the network obsolete. Most of the efficient routing protocols such as LEACH, SEP, T-DEEC, MODLEACH, EESAA, genetic HCR and ERP, *etc.*, work on optimizing the energy efficiency, but they do not consider the other factors like delay, load balancing, scalability, *etc.* Their efficiency decreases as the network size increases so they are not suitable for environmental monitoring, military or real time IoT applications. Moreover, the energy scarcity of low price and low-powered sensor node has been a central issue for WSNs and for the future IoT. To extend its lifespan, the sensor nodes operate in a duty-cycled mode. The recent development of energy harvesting technologies mitigates the energy scarcity issue, but the sensor node still has to operate in duty-cycled mode due to the limited energy collection from the environment (e.g., light, RF, and vibration), and has to dynamically adjust its duty cycles to accommodate the availability of environmental energy. Such dynamic duty cycles pose challenges for networks with IEEE 802.15.4 MAC in terms of synchronization, packet loss, waste of channel resources and energy, and so on, therefore, standards for duty-cycling-aware middleware between MAC and power management are highly desired [62].

Based on the above discussed challenges, requirements and advances, it is identified that existing schemes lack in providing the energy efficiency, reliability, scalability and timely delivery required for WSN-based IoT applications. With this objective in mind, we propose ME-CBCCP, a chain-based routing algorithm for homogeneous WSN/IoT scenarios.

## 3. System Model and Framework

### 3.1. System Architecture

Complexity in the IoT is higher than in WSNs, as it has a large number of objects and due to this reason it has a large scale. Dynamic routing (a routing scheme in which routes can be changed at any time during data transmission or routes are decided during the processing of data as proposed in [11,13,17,63]) for WSN architectures is not suitable in large scale areas, e.g., in environment monitoring applications. The transmission of sensors is affected by many factors like air humidity, temperature and interference, so WSN architectures with dynamic routing are unusable for large scale networking. Tiny sensor nodes have constraints of low battery energy, low power and low memory and in large scale applications their processing requirements are increased. Sensor nodes require information regarding their location to be exchanged in dynamic routing. Consequently, nodes consume more power due to the increased overhead. That is why this type of routing is not applicable to the IoT. Moreover, network components deployed in the IoT are less mobile and their topology remains stable, so a dynamic routing configuration does not gain much over static routing.

**Figure 1:**
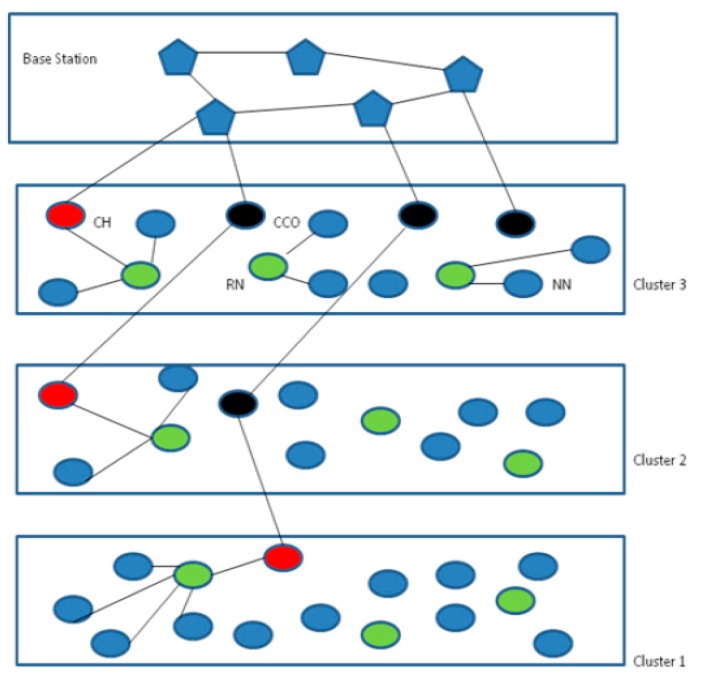
Multi-tier Framework for IoT (CH: Cluster Heads; CCO: Cluster Coordinators; RN: Relay Nodes; NN: Normal Nodes).

Considering the above factors we propose static and energy efficient routing for a scalable and complex IoT. We studied the effect of using our transmission algorithm over a thousand nodes deployed in a 200 and 300 m^2^ area with different numbers of nodes and found that static routing is really suitable for scalable IoT applications. We have used the same tiered framework as used in [1], except for the relay layer which is not used in our framework, as can be seen in Figure 1. It presents the hierarchical network structure where all objects placed are static and follow the transmission based on static routing.

The lower layers consist of sensor nodes, cluster heads, relay nodes and cluster coordinators. The topmost layer is the convergence layer. This layer is comprised of base stations which are connected to the internet. In the lower layers, nodes sense the thing or the objects and transmit the data to the RN nodes. RNs pass the data to the CHs. To balance the load on the CHs and CCOs, CHs pass the data to the upper layer CCO which further hands over the data to the upper layer CCO and this process continues till the data is transmitted to the BS at the topmost layer. To conserve energy, nodes are not allowed to communicate with each other within the cluster and with the upper layer cluster. Locally, information is transmitted via RNs and communication with neighbor cluster is through the CHs and CCOs only. This deployment supports the energy efficient and scalable IoT. This type of deployment is general and can be used for any type of application environment monitoring, border surveillance, *etc.*

This scheme is flexible, provides scalability and hence can be easily managed. By the placement of IoT components above this framework, energy can be conserved in an efficient manner. As routing is static and simple, soon advantage of this layered scheme is that the various components of the IoT do not need complicated hardware and complex methods, which is very cost effective strategy.

Let there be in the Euclidean plane two points u and v, and let the distance between them be *d*(*u,v*), the set of the local nodes within the cluster is denoted by NN1, the set of cluster coordinators is denoted by CC1, the set of relay nodes is denoted by RN1, the set of base stations is denoted by BS1, the set of CHs is denoted by CH1, the set of cluster ids is CLS_IDand the lower cluster is denoted by L_CLS and the upper cluster is denoted by U_CLS. A network of the IoT is denoted by G(N,V), where N is the set of all the nodes between the field and V is the set of wireless links among the nodes. R is the communication radius of the relay nodes and *r* is the communication radius of the local nodes where *R ≥ r > 0*. The data transmission or communication policy between two nodes in this network can be summarized as below:

#### 3.1.1. Communication within Clusters

Communication is not possible when:

(1) if u ϵ NN1, v ϵ NN1 and d(u,v)≤r
(2) if u ϵ NN1, v ϵ CH1 and d(u,v)≤r
(3) if u ϵ RN1, v ϵ RN1 and d(u,v)≤R

Communication is possible with the following conditions:

(4)  if u ϵ NN1, v ϵ RN1 and d(u,v)≤r
(5) if u ϵ CH1, v ϵ RN1 and d(u,v)≤r

(nodes can communicate to CHs via RNs).

#### 3.1.2. Communication among Clusters

Communication is not possible in the following conditions:

(1)  if u ϵ NN1 in L_CLS, v ϵ NN1 in U_CLS and d(i,j)≤r
(2) if u ϵ NN1 in L_CLS, v ϵ RN1 in U_CLS and d(i,j)≤r
(3) if u ϵ RN1 in L_CLS, v ϵ RN1 in U_CLS and d(u,v)≤R
(4) if u ϵ NN1 in L_CLS, v ϵ CH1 in U_CLS and d(u,v)≤r
(5) if u ϵ CH1 in L_CLS, v ϵ CH1 in U_CLS and d(u,v)≤r
(6) if u ϵ RN1 in L_CLS, v ϵ CH1 in U_CLS and d(u,v)≤r
(7) if u ϵ CH1 in L_CLS, v ϵ CH1 in U_CLS and d(u,v)≤r

Communication is possible in the following conditions only:

(1) if u ϵ CH1 in L_CLS, v ϵ CC1 in U_CLS and d(u,v)≤r, and CLS_id (L_CLS) =CLS_id+1 (U_CLS)
(2) if u ϵ CC1 in L_CLS, v ϵ CC1 in U_CLS and d(u,v)≤r, and CLS_id (L_CLS) =CLS_id+1 (U_CLS)
(3) if u ϵ CC1 in L_CLS, v ϵ BS in U_CLS and d(u,v)≤r, and CLS_id (L_CLS) =CLS_id+1 (U_CLS)

With these symbols and notations, we have made the following assumptions of the system model.

(a) The sensor nodes are stationary and symmetric. Nodes can communicate using the same transmission power level that means links are symmetrical and each node is allocated a unique ID .The importance and potential of all the nodes are equal to extend the lifetime of the network.
(b) Routing techniques are required to balance the energy consumption among the nodes because the network supplies information to stationary observers positioned at the border of the area, which entails that energy consumption is not uniform for all nodes.
(c) The number of transmission power levels are fixed for each node.
(d) Nodes are equipped with GPS-capable antennae. The location of the nodes is tracked in the initial phase after that GPS will be turned off because the nodes are not mobile.
(e) It is not possible to recharge the batteries of the nodes as they are left unattended after deployment. Hence there is requirement of energy efficient routing protocols.
(f) Data is transmitted to the BS in multi-hop manner and BS layer are not constrained in energy while the other layers have the constraint of limited energy.
(g) The network of IoT is fully connected as BS is reachable to each and every node.

In Section 4, an energy efficient IoT is modeled based on the assumptions discussed here.

### 3.2. Research Problem Foundation

From the literature survey discussed above in Section 2, we found that a routing protocol in WSN/IoT may face the problems: (1) due to long path communication; (2) due to failure of some nodes; (3) due to latency in the delivery of data; (4) protocols developed for small scale applications break down when the number of nodes is increased; (5) due to load imbalance over the CHs; (6) due to single hop communication, *etc*. To deal with these problems, many protocols have been proposed in the recent past. With LEACH [28], election of the optimum cluster heads reveals that it can improve the network lifetime, but direct communication of the nodes with the CHs poses a problem, so many other protocols have been proposed to date to improve the clustering scheme as proposed by LEACH. SEP [29] improved the network lifetime as compared to LEACH by exploiting the benefits of heterogeneity. TDEEC [39] also tried to increase the stability period of the network by using multi-level heterogeneity and by considering the threshold value of energy during the election of CHs. EESAA [40] has taken into account one more parameter in addition to residual energy and that is switching between sleep and active modes to save energy. By using the dual power mode that is low power for intra-cluster communication and high power for inter-cluster communication, MODLEACH makes an attempt to minimize the energy usage of the nodes.

Clustering-based genetic protocols such as genetic HCR [46], ERP [45] improve the stability period for the working of the WSN as compared to SEP and LEACH by the election of new CHs based on mutation, but these protocols take a lot of time in the election of the best breed for the CHs.

From these protocols it is concluded that clustering is the best topology to maximize the energy efficiency, which is the stringent requirement of IoT applications and clustering facilitates the scalability feature for the IoT which is required due to its increased network size. Less processing of the nodes can also further enhance the energy level. As in the above protocols CHs are elected in every round based on different strategies such as the optimum number of CHs, based on residual energy and based on mutation. This gave us the idea that instead of the election of CHs again and again in each round, the role of CHs can be exchanged with the high energy nodes only when it is found in some round that the energy of some CHs has fallen below some threshold level. The disadvantages of direct communication of the nodes with BS gave us the idea to use multi-hop communication which is made possible by the chain method as used in [53] and it also assures the connectivity of the nodes (although only a fully connected network is assumed). A routing protocol, which works well even when the number of nodes and area is increased is the most suitable for an energy efficient IoT. Problems (1–6) discussed above motivated us to develop a new protocol which could cater to the needs of an efficient IoT. Development of the energy efficient IoT is the challenging issue and its complexity is increased as protocols already developed for WSN a cannot be applied directly to it. The main contributions of this paper include: (1) a hierarchical framework for the IoT; (2) a model for an optimized solution on the basis of the proposed framework for the IoT; (3) a minimum energy consumption algorithm for the optimized model.

## 4. Model for an Energy Efficient IoT

With the above discussed framework and research problem, the main objective of ME-CBCCP is to reduce the communication distance between all the objects to compensate the power consumption in the lower layers of the IoT framework. In this section we have explored the various notations (Table 1) used in this paper and system constraints are formulated as per the requirements of an energy efficient IoT. Next, we address the optimization problem of an energy conserved IoT. To minimize the energy consumption in this framework, a minimal energy consumption transmission algorithm is proposed as a solution to the optimization problem.

### 4.1. Constraints Imposed in IoT System

A normal node can communicate only in a local location that is, it can only transmit data to the relay node whose distance is minimized from that node. Normal nodes can only transmit the data but relay nodes and cluster head nodes can perform both the tasks of transmission and reception of data. In local cluster communication, RNs, CHs and NNs can communicate with each other, but the CH of the local cluster can communicate with the CCO of the upper cluster whose cluster id is one greater than the lower cluster of that CH. CCO of the lower layer of the uppermost layer can transmit and receive data from the BS layer. Because of the abovementioned constraints, G(N,V) is known as a connected and directed graph. If two nodes *u* and *v* can communicate with each other, they are known as the neighbor nodes. Let *N(u)* be the set of *u’*s neighbors and *R* be the adjacency matrix of G(N,V):

(1) $$\text{P}=[\begin{array}{cccc} r_{11} & r_{12} & .. & r1_{N}\\ r_{21} & r_{22} & .. & r2_{N}\\ . & . & . & .\\ r_{\left|N\right|1} & r_{\left|N\right|2} & r_{\left|N\right|.} & r_{\left|N\right|\left|N\right|} \end{array}]$$

where P(u,v) = 1 if v∈N(u) otherwise P(u,v) = 0. The system constraints for IoT requirements are as follows:

**Table 1:** Parameters used in ME-CBCCP for IoT.

$E_{Tx}$	Energy Consumption for data transmission
$E_{Rx}$	Energy Consumption for data reception
$E_{elec}$	Energy Consumption for radio electronics
$\in_{1},\in_{2},\in_{3},\in_{4}$	Transmit amplifier of the normal node, relay node, and CH node and CC node respectively
x,y,z,p,q	Cardinality of NN1, RN1, CH1, CC1 and BS1
$S_{\left(u,v\right)}$	Data rate from node u to v
$S_{\max}$	Maximum data rate
ℓ	Data length of the packet
$C_{cc}$, $C_{ch}$, $C_{rn}$, $C_{bs}$, $C_{nn}$	Cost of CC, CH, RN, BS and NN
|.|	Cardinality of a set
$F_{0}$	System budget
$d_{\left(u,v\right)}$	Distance from node u to v

### 4.2. Energy Expenditure Constraints

The energy depletion in processing and sensing is muchless as compared to data transmission and reception. The energy depletion in data communication is taken into account according to the Friis space model [64]:

(2) $$E_{Tx}=\ell \;\times \;{\text{E}}_{\text{elect}}+\ell \;\times {\text{E}}_{l}\;\times d^{4}$$

(3) $$E_{Rx}=\ell \times {\text{E}}_{e}+\ell \times {\text{E}}_{bf}$$

(4) $$E_{Tx}=\ell \times {\text{E}}_{e}+\ell \times {\text{E}}_{s}\times d^{2}$$

*E_l_* is used for amplifier of NN, RN, CH and CC nodes and in the manipulation of the equations they are used as $\in_{1},\in_{2},\in_{3},\in_{4}$ respectively. Equation (2) is used for long distance communication and Equation (4) computes the energy depletion in the short distance communications. Data is received at relay nodes, CH nodes and cluster coordinator nodes so the energy released in this task is computed by Equation (3). The data rate for the nodes u to v is the same as the data length *l*. Energy depleted by each node per unit time is computed by:

(5) $$E_{u}=\sum_{v\in RN}r_{uv}\cdot S_{uv}\cdot ({\text{E}}_{elec}^{NN}+\in_{1}\cdot d_{u,v}^{2})\forall \text{u}\in NN$$

(6) $$E_{v}=\sum_{u\in NN\cup RN}r_{uv}\cdot S_{uv}\cdot {\text{E}}_{elec}^{RN}+({\text{E}}_{elec}^{NN}+\in_{2}\cdot d_{uv}^{2})+\sum_{u\in BS\cup RN}r_{vu}\cdot S_{vu}\cdot ({\text{E}}_{elec}^{RN}+\in_{2}\cdot d_{vu}^{2})\forall \text{v}\in RN$$

(7) $$E_{x}=\sum_{u\in RN\cup CH}r_{vx}\cdot S_{vx}\cdot {\text{E}}_{elec}^{CH}+({\text{E}}_{elec}^{CH}+\in_{3}\cdot d_{vx}^{2})+\sum_{u\in CH\cup CCO}r_{xv}\cdot S_{xv}\cdot ({\text{E}}_{elec}^{CH}+\in_{3}\cdot d_{xv}^{2})\forall x\in CH$$

(8) $$E_{y}=\sum_{u\in CH\cup CCO}r_{xy}\cdot S_{xy}\cdot {\text{E}}_{elec}^{CCO}+({\text{E}}_{elec}^{CCO}+\in_{4}\cdot d_{xy}^{2})+\sum_{u\in CCO\cup BS}r_{xy}\cdot S_{xy}\cdot ({\text{E}}_{elec}^{CCO}+\in_{4}\cdot d_{xy}^{2})\forall y\in CCO$$

(9) $$E_{z}=\sum_{y\in CCO}r_{yz}\cdot S_{yz}\cdot {\text{E}}_{elec}^{BS}\forall z\in BS$$

$E_{u},E_{v},E_{x},E_{y},E_{z}$ denote the energy consumed by sensor nodes, relay nodes, cluster head nodes, cluster coordinator nodes and base stations, respectively, in the transmission and reception processes. The symbols $E_{elec}^{NN},E_{elec}^{RN},E_{elec}^{CH},E_{elec}^{CCO},E_{elec}^{BS}$ denote the energy consumed by the electronics of a normal node, relay node, cluster head node, cluster coordinator node and base station node. Equation (5), shows the energy depletion in data transmission by a normal node to the relay node within the local cluster. Equation (6) is used to compute the energy consumption in the process of data reception and transmission by the relay node to the CH and Equation (7) shows the energy depletion by CH in data communication with the upper layer cluster coordinator. Equation (8) depicts the energy used for the data communication process by a cluster coordinator with the upper layer cluster coordinator or the BS. Equation (9) depicts the energy consumed by the base stations in the BS layer. In these equations, energy consumed in transmitting data by the BS to the nodes is excluded because the data received by the nodes are the signaling data [1] and it consumes much less energy.

### 4.3. Wireless Links Constraints

In the framework of the Internet of Things, the topmost layer which is composed of base stations provides the connectivity of the all the BS using wired links. Hence, the bandwidth of the BS layer is greater than that of all the nodes in the lower layers. The relay nodes transfer the data to the CHs so constraints on the wireless links of RN are given by Equation (10):

(10) $$r_{uv}\cdot S_{uv}\leq S_{\max}\forall u,v\in RN\cup NN$$

The constraints on the wireless links of CCO, CH and BS are shown in Equation (10); they have communication with lower and upper layers so both the tasks are considered in this constraint:

(11) $$r_{xy}\cdot {\text{S}}_{xy}+r_{yx}\cdot S_{yx}\leq S_{\max}$$

where ∀x∈RN, ∀y∈CH or ∀x∈CH, ∀y∈CCO or ∀x∈CCO, ∀y∈BS.

Nodes nearest to the BS deplete their energy faster than other nodes as they transmit the data to BS on the behalf of other nodes. Thiscan partition the network. To avoid this problem, we propose a hierarchical IoT framework so that the load should be balanced on the nodes (RN, CH and CCO). Each RN transmits the data of the local nodes which have a minimum distance from them. CHs and CCOs have the load of the nodes of one cluster only. Thus load balancing on their wireless links support an energy efficient IoT.

### 4.4. Optimization Problem for Energy Efficient IoT

The main objective of this paper is to enhance the network lifetime to achieve an energy efficient IoT, so the optimization for IoT based on the above constraints can be modeled as:

(12) $$T_{E}=\min\left[\sum_{u\in NN}E_{u}+\sum_{v\in RN}E_{v}+\sum_{x\in CH}E_{x}+\sum_{y\in CCO}E_{y}\right]$$

Equations (5)–(10) indicate that the optimization problem is to reduce total energy consumption, *T_E_*. There are other issues for efficient IoT-like robustness, and timely delivery of data which are covered in this paper with the help of ME-CBCCP, but the first and foremost requirement is to minimize the energy depletion of the nodes which are performing various roles.

### 4.5. System Budget Constraints

As deployment of various objects in the IoT framework is very expensive, one requirement is to keep the budget low as estimated initially in Equation (13). It can be considered during the hardware installation of the components, but this requirement is out of the scope in the recent work proposed in this paper:

(13) $$\sum C_{cc}+C_{ch}+C_{rn}+C_{nn}+C_{bs}\leq F_{0}$$

**Theorem 1.** *The problem in Equation (12) is a NP-hard problem*.

**Proof of Theorem 1.** To find the route to solving the Equation (12) is a NP-hard problem like the travelling salesman problem as it tries to find out the least cost cyclic route in weighted graphs. As we want to establish an energy efficient IoT framework, we require minimizing the energy consumption in the whole network, so we develop the ME-CBCCP to reduce the energy consumption by balancing the load on the CHs and CCOs which act as the destinations (including BS which have unlimited supply of power) for the normal nodes.

The basic idea behind this algorithm is to use clustering topology on the basis of division of the area into same sized rectangle shaped cells which are known as layers of clusters. ME-CBCCP follows five steps to accomplish the main objective:

Step 1, suggests the deployment of thousands nodes randomly in the fixed area.

In Step 2, ME-CBCCP employs the method of dividing the area into same size cells with fixed boundaries. For example, if the area is 200 m^2^, then each subarea is about 200 m by 20 m and there will be 10 clusters. Within a cluster, the normal nodes (NN1) select the closest relay nodes and form different groups under different relay nodes (RN1). Relay nodes are formed randomly and the percentage of relay nodes within the cluster is 0.1. Other nodes (0.9) act as the normal nodes.

ME-CBCCP elects one CH and a varied number of CCOs in each cluster in Step 3. The energy of each CH and CCO is checked, and should be greater than the threshold energy. The distance between the relay nodes and normal nodes is checked and if it is less than or equal to the communication radius, then that node is added to the set of nodes under that relay node.

In Step 4, the data transmission starts from normal nodes to the relay nodes, which is further transmitted to CH and then via CCOs, it is finally submitted to the BS in the topmost layer.
**Algorithm 1. **Minimum Energy Consumption Chain Based Algorithm (ME-CBCCP)
**Input**

NN,RN,CH,CCO,BS, R>r>0

**Output**

**Enhanced network lifetime **

**1: Deploy nodes randomly in the fixed area**

**2: Apply subarea division algorithm to form the clusters (**number of clusters=k**) with fixed boundaries, allocate the cluster IDs to each layer of the cluster**
cls_id<=k**. That is for**
∀NN∈N, NN1∈Local_cluster** Select the closest **RN1** within cluster **u ϵ RN, v∈NN1**.**
**3: for each **cls_id<=k**, repeat**
**3.1 Elect one **CH** in each cluster randomly **x∈CH** where **x.energy>threshold.energy**. Elect **CCO** in each cluster where **y∈CCO** and **y.energy>threshold_energy** and value of **0<y<cls_id−1** and. Number of C **CCO** in each cluster = **cls_id−1**.****3.2 for **u ϵ RN, v∈NN1**, **u≠v ** and **u.energy and v.energy > 0** do****3.3 compute the distance **${\text{d}}_{\text{uv}}$** between **u** and **v**3.4 if **${\text{d}}_{\text{uv}}\leq R$** then****3.5 add the node **u** and **v** to the set of **RN1** in local cluster, set **P(u,v)=1**   End if****   End for**
**End for**

**4: for each **cls_id<=k**, repeat**
**4.1 transmit the data from **v** nodes (**v∈NN1**) to **u** nodes (**u ϵ RN1**), ****4.2 transmit data in order from **j→x→y→z** nodes **(x∈CH,y∈CCO,z∈BS )
**End for**

**5: for each **cls_id<=k**, repeat**
**5.1 Compare: if **x.energy and y.energy≤threshold.energy** Then****Go to step 3.****Else**
**Continue with step 4.**
**End if**
**End for**



As the network grows in size, it becomes difficult to maintain its reliability, but all the applications require that the network should not be partitioned and the transmission algorithm should be robust. To make ME-CBCCP more suitable for IoT applications, in Step 5, the energy level of each CH and CCO is checked, and if it is found that their energy level is far less than the required level to act as the CH and CCO, then their role is changed by reelecting CHs and CCOs by moving back to Step 3. In this way the transmission process will continue even when some nodes have depleted their energy up to the largest extent.

Equation (12) presents the optimization problem, by which the energy consumption in each cluster should be decreased. This is achieved by balancing the load on the clusters by a cooperation method among the nodes, CHs and CCOs. Moreover ME-CBCCP is a static algorithm in contrast to the traditional dynamic algorithms of WSNs. In dynamic algorithms, nodes exchange their information about the routes during the network operation and this increases the network overhead which is why those algorithms cannot be applied to IoT framework.

**Theorem 2.** *The clusters are formed in fixed iterations and the complexity is O (1)*.

**Proof of Theorem 2.** According to Algorithm 1, in Step 2 the clusters are formed by division of the area into subareas which are fixed. Due to static clustering, this process is accomplished in one round only. For NN1 nodes the probability of election as the CH is much less during the initial phase that is 1/NN1. With the increase in the number of dead nodes (d), the CH _ prob also increases. CH _ prob=1/NN1−d, where 0 <= d < NN1. Nodes with more energy than the threshold energy can be CH and nodes with less energy than the required energy will join the cluster as normal nodes. As the number of dead nodes increases, the number of iterations decreases (NN_ itr α 1/NN1−d).

**Theorem 3.** *The complexity for N nodes for message exchange is O (N)*.

**Proof of Theorem 3.** According to Theorem 2, the number of iterations is fixed for clustering and for electing CHs. So it takes, at most, N time for their processing. Computation of the total time taken in the data communication process can be considered as N _ ITR×N (for the transmission) of data of N nodes in N _ ITR). Hence the complexity to exchange the messages in N nodes is taken as O (N).

**Theorem 4.** *Each rectangle cell has at least CH to aggregate the data from local nodes*.

**Proof of Theorem 4.** Data is aggregated from the local nodes by CHs in each cell. From Step 3 in Algorithm 1, each cluster will have one CH. In the worst case, if a cluster has only one node, then it will act as the CH till its energy falls below the threshold level. It will coordinate with the upper layer CCO in transmitting the data. If there is no CCO in the upper layer (due to depletion of energy of the nodes by time) then data is transmitted directly to the BS to avoid delay in transmitting the data which are the requirement of many applications of IoT.

The network lifetime of ME-CBCCP can be easily computed. The network lifetime can be considered in various ways. For example, it can be considered as the time period from the initial operation till the death of first node or it can be considered as the time span from initial operation of the death of all the nodes. We have considered the second option. To provide an in-depth insight for the whole IoT, the worst case of network lifetime can be given as:

(14) $$N^{l}=\min\left(\frac{E_{1}}{E_{u}},\frac{E_{2}}{E_{v}}\frac{E_{3}}{E_{x}},\frac{E_{4}}{E_{y}}\right)$$

where $E_{1},E_{2},E_{3},E_{4}$ defines the initial energy of normal nodes, relay nodes, cluster heads and luster coordinators.

With the use of RNs, CHs and CCOs the network is connected and an energy efficient IoT can be easily deployed with this scheme.

## 5. Performance Evaluation and Results Discussion

### 5.1. Setup Phase

**Figure 2:**
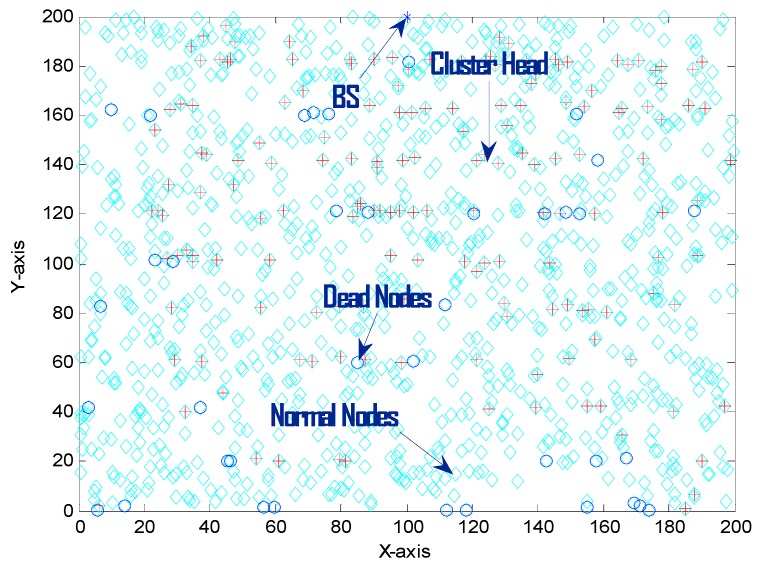
Smulation after 200 rounds of data transmission.

The validation of effective deployment of the proposed strategy is presented in this section via numerical computations. The nodes are completely distributed in the 200 × 200 m^2^ field. In Figure 2, in a randomly formed topology, the percentage of relay nodes is 0.1 and for ten layers of clusters there are 10 CHs and 45 CCOs. $E_{elec}$ for all the nodes is 50 nJ/bit. $E_{l}$ for long distance the energy consumed is 0.0013 pJ/bit/m4 and $E_{s}$ for short distance is 10 pJ/bit/m2. Energy used in beam forming $E_{bf}$ is 5 nJ/bit. The value of N is 1055 for 200 m^2^ and 1500 for 300 m^2^. The length ℓ of data is 4000 bits. For a scalable and energy efficient IoT, we considered two scenarios one with the value of N, 1055 for 200 m^2^ area and other with value of N, 1500 for 300 m^2^ area. The initial energy of all the nodes is 0.5 J.

### 5.2. Results Discussion

**Figure 3:**
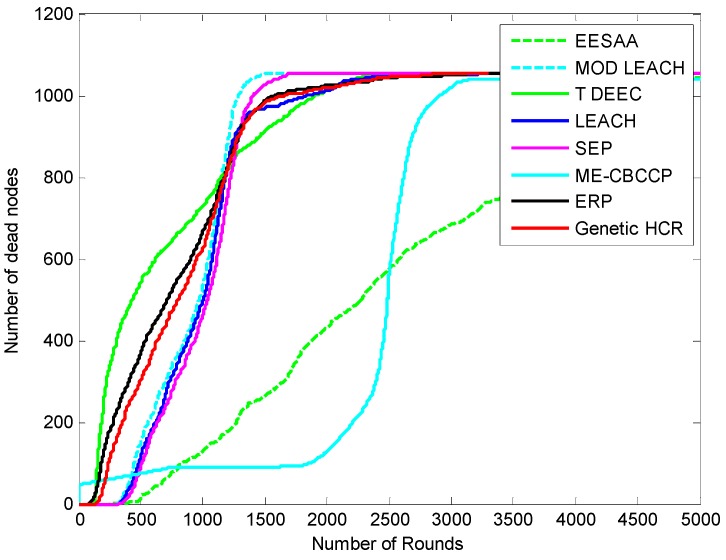
Network lifetime comparison (1000 nodes, with the help of alive nodes, 200 m^2^).

**Figure 4:**
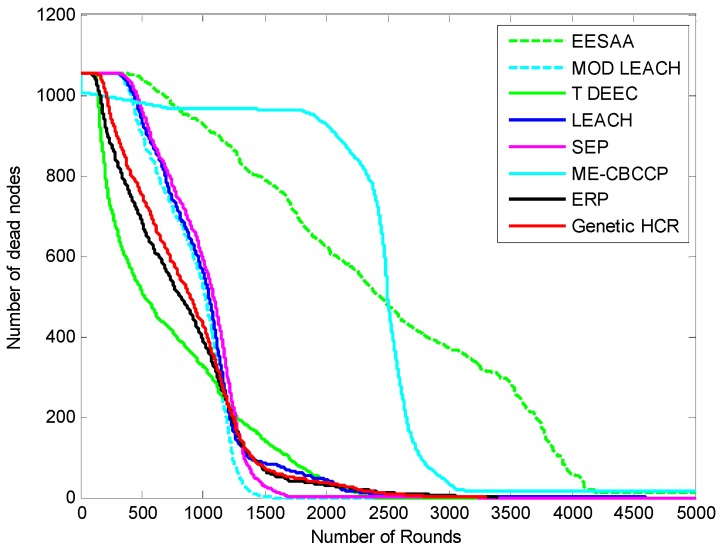
Network lifetime comparison (1000 nodes, with the help of dead nodes in 200 m^2^).

**Figure 5:**
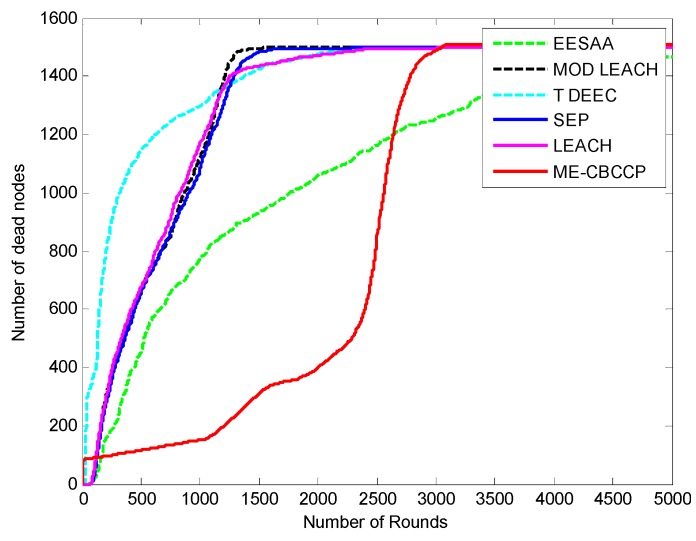
Network lifetime comparison (1500 nodes, with the help of alive nodes in 300 m^2^).

**Figure 6:**
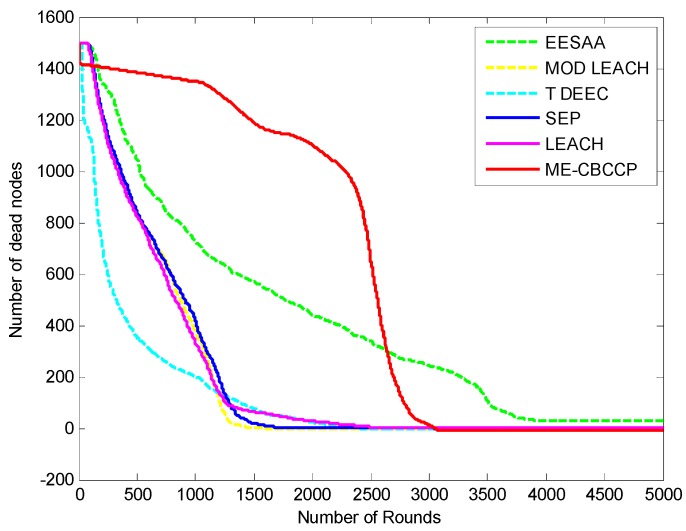
Network lifetime comparison (1500 nodes, with the help of dead nodes in 300 m^2^).

The results discussed here are measured after approximately 95 simulations. Figure 2 shows the network after 200 rounds of data transmission. The number of CHs, relay nodes and CCOs will increase with the increase in the scale because with the increase in the size of the network, so a large number of coordinators will be required to connect and maintain the cooperative process. When the position of the BS and other nodes (RN, CH, CCO) is fixed, then less energy is consumed as there is no need to search again and again for these nodes. If the communication radius of the nodes is increased they can cover a large area, and then these nodes can be decreased in number in each layer. Nodes are deployed randomly, so their density is high. The number of nodes required for the cooperative process can vary according to the density of the nodes and scale of the network for energy efficient IoT. Figure 3, Figure 4, Figure 5 and Figure 6 show the network lifetime for different scales of the Internet of Things. These figures show the comparison of network lifetime of the network with different traditional protocols proposed for WSNs in the recent past.

### 5.3. Comparison of Network Lifetime

The protocols discussed in this paper are proven to be energy efficient with cluster topology. The assumption of a sink in the center is the best method to aggregate the data as the distance for the nodes will be reduced, but various applications (border security, environmental monitoring, *etc.*) of the IoT do not consider this assumption. The IoT considers the various objects installed at the application layer where a user can interact and collect the data. This layer should have an unlimited power supply to collect data from various regions, so we have assumed this condition by forming the topmost layer consistingof the base stations which are interconnected and have unlimited power. Figure 3 shows the number of rounds *versus* the number of the dead nodes graph. It can be noticed that all nodes are dead in LEACH after 3200 rounds (transmission of data by all the nodes completes one round), in ERP after 3370 rounds, in genetic HCR after 3305 rounds, in SEP after 3900 rounds, in MODLEACH after 1590 rounds, and in T-DDEC after 2401 rounds, but in EESAA 11 nodes are alive after 5000 nodes, andin ME-CBCCP 15 nodes are alive.

To make it more clear, Figure 4 depicts the dead node graph after 5000 rounds. After 500 rounds of data transmission, 90 nodes are dead in SEP, 113 in LEACH, 368 in ERP, 303 in genetic HCR, 121 in MODLEACH, 543 T-DDEC, 14 in EESAA and 49 nodes in ME-CBCCP. After 1000 rounds of data transmission, SEP, LEACH, ERP, genetic HCR, MODLEACH, T-DEEC and EESAA lost 459, 493, 660, 625, 535, 726, and 129 of their nodes, respectively, but only 73 nodes are dead in ME-CBCCP. This shows that ME-CBCCP is an optimal solution for an energy efficient IoT.

We increased the network scale and number of nodes to know about its efficiency for the IoT, and it is found that with the increase in the number of nodes and network area, ME-CBCCP still performs better as can be noted from Figure 5 and Figure 6.

### 5.4. Comparison in Delay

SEP, LEACH, EESAA take 29%, 22.22% and 1423% more time in transmission when compared with ME-CBCCP, but with the increase in the number of nodes, genetic protocols, HCR and ERP took nearly 23 and 25.5 h to execute.

**Figure 7:**
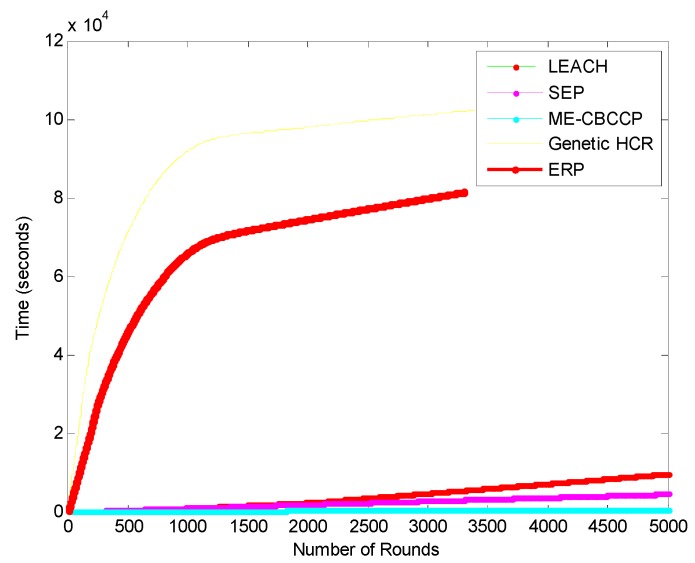
Transmission delay (I) (1000 nodes, 200 m^2^).

ME-CBCCP performs computations within and outside the cluster level so when we compared it with MODLEACH and T-DEEC it takes 20% and 7% more time than both, but this is tolerable due to the better gain in terms of parameters, network lifetime and scalability as can be noticed from Figure 7. The total time taken in transmission in ME-CBBCP is 537 s, but SEP and LEACH take 1700 s and 3400 s, respectively, to execute.

**Figure 8:**
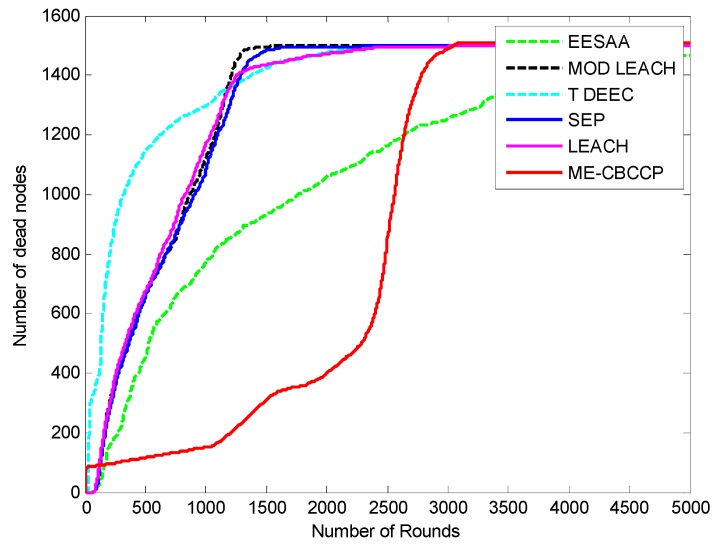
Transmission delay (II) (1000 nodes, 200 m^2^).

**Figure 9:**
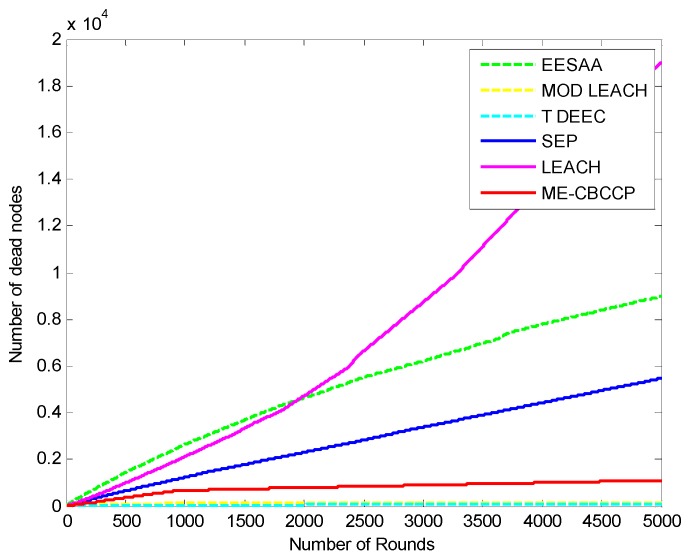
Transmission delay (1500 nodes, 300 m^2^).

MODLEACH takes 275 s, T-DEEC takes 464 s and EESAA takes 8000 s in execution (Figure 8). In Figure 9, the results of transmission delay are shown in a large network and with the number of nodes. Results show that ME-CBCCP is performing better than other protocols. It is proved by these results that traditional protocols for WSNs cannot be applied to the IoT framework directly and they need to be modified.

### 5.5. Comparison in Scalability

ME-CBCCP is scalable so when the number of nodes and dimensions of the area are increased, even then it shows its improvement over other protocols. The comparison of ME-CBCCP with a number of nodes (1500) with same numbers of cluster heads in the area of 300 m^2^ is performed. It is observed that it outperforms LEACH, SEP, genetic HCR, ERP, MODLEACH, T-DEEC in terms of network lifetime and stability and LEACH, SEP, genetic HCR, ERP, EESAA in terms of time. Genetic HCR and ERP are not suitable for the dense network as around 500 nodes are dead after 450 rounds and they again took 26 to 29 h to execute. Both of these protocols are not comparable in this simulation so we excluded them in this comparison. In the parameter of network lifetime the result of EESAA and ME-CBCCP is comparable as EESAA lost 456 nodes in 500 rounds but ME-CBCCP lost only 123 nodes. In 1000 rounds ME-CBCCP lost 162 nodes, but EESAA lost 776 nodes. However, after 5000 rounds no node is alive in ME-CBCCP but 25 nodes are still alive in EESAA. This happens because the subarea which is assumed as one cluster in ME-CBCCP has dimensions of 200 m by 20 m, but in the simulation of 1500 nodes in the area of 300 m^2^ the number of clusters are kept same, that is only 10 clusters and one cluster has dimensions of 300 m by 30 m, so the distance of transmission is increased for the nodes which results in the death of the number of nodes after 2500 transmissions. Before that it works better than EESAA in terms of network lifetime. This can be solved by getting back to Step 2 of Algorithm 1. In terms of the time parameter ME-CBCCP again outperforms EESAA.

### 5.6. Analysis of ME-CBCCP in Terms of Other Parameters

If the residual energy of the nodes is considered during the execution of the routing protocols and the role of the CH is replaced by high energy nodes then connectivity of the nodes can be maintained. This can assure the transmission of the data even in the case of failure of some nodes. This aspect is required to make the routing protocol for the IoT framework more reliable, but this fact has not been taken into account in LEACH and SEP. MODLEACH, TDEEC and EESAA. ERP and genetic HCR are based on the election of the best class of CHs so they withstand well in terms of fault tolerance. This feature enhances the stability period of the protocols. ME-CBCCP also considers the residual energy of the nodes and the role of CHs with less energy is exchanged with the other (high energy) nodes in the next round. Load balancing on the nodes is achieved by nodes with the help of CHs and CCOs. CHs gathers the data of one cluster only and CCO dedicated to one cluster will always transmit the data of that particular cluster. In this way CHs and CCOs will have an approximately the same load that is the loads of one cluster only.

From the above discussion, it is clear that ME-CBCCP successfully optimized the most stringent constraints of the WSN for an energy efficient and scalable IoT, that are time and energy. However,this novel protocol works only for large scale networks. It works well for static nodes. A static algorithm can cause problems in a dynamic environment (although failure of nodes due to energy has been considered in this protocol). This routing algorithm can suggest new directions for future work because quality of service (QoS) parameters can be enhanced further by using mobile nodes or mobile sinks to gather the data but mobility is not appropriate for IoT applications so it is a better option to optimize it in the static mode. The dumb behavior of the nodes can affect the workings of the stationary network. Nodes which can have an adverse effect on the performance of the WSN can be avoided in regular operation which can make ME-CBCCP more effective.

From the abovementioned points and comparisons it is clear that the network lifetime of the IoT in a layered architecture (hierarchical) is longer than that of other schemes. In other schemes the relay nodes nearest to the BS can be overloaded and their energy will deplete earlier than that of other nodes. Imbalance of the load causes the nodes to deplete their energy earlier and hence network partition can occur, but with a cooperative method which reduces the communication distance, energy efficiency has been increased and it is suitable for the IoT. With the deployment of CHs, RNs and CCOs energy efficiency has been increased which would prolong the network lifetime of the IoT. Therefore, we assert that proposed hierarchical strategy is preferable for an energy efficient IoT.

## 6. Conclusions and Future Scope

Energy efficiency schemes have a significant role in developing an efficient IoT. In this paper, we have proposed an optimized solution to the problem of arranging objects in the implementation of an energy efficient and scalable IoT. Firstly, we gave the framework for the deployment of the IoT which has scalability features and makes it more extensible. Then, based on the framework, an optimization scheme which is constrained by the loads on wireless links and energy expenditure support the deployment of an energy efficient IoT. Lastly the ME-CBCCP algorithm has been proposed based on the clustering topology as a solution to the optimization problem. With numerical experiments, it is validated that the proposed scheme is better than traditional WSN schemes in terms of time, network lifetime and scalability. It is found that that new scheme consumes six times less energy than LEACH, five times less energy than SEP, four times less energy than genetic HCR, three times less energy than ERP, three times less energy than MODLEACH, two times less energy than T-DEEC and 1.4 times less energy than EESAA which further validate our work. It takes 23.22% less time than LEACH, 29% less time than SEP, 152 times less than ERP, 229 times than genetic HCR, 15% less time than EESAA, and 2 times less time than MODLEACH. In the future we will also try to exploit the benefits of heterogeneity and we will also propose improvements of end to end delay, data compression techniques, packet delivery ratios and throughput parameters, to achieve a more efficient green IoT.
